# Finite Element Analysis of Extrusion Process for Magnesium Alloy Internal Threads with Electromagnetic Induction-Assisted Heating and Thread Performance Research

**DOI:** 10.3390/ma13092170

**Published:** 2020-05-08

**Authors:** Meng Liu, Zesheng Ji, Rui Fan, Xingguo Wang

**Affiliations:** 1 Departments of Materials Science and Engineering, Harbin University of Science and Technology, Harbin 100080, China; liumeng5201@126.com; 2 Departments of Mechanical and Electrical Engineering, Qiqihar University, Qiqihar 161006, China; fanrui_2325@163.com (R.F.); wangxingguo2014@163.com (X.W.)

**Keywords:** electromagnetic induction, DEFORM-3D, Material flow, hole diameter, heating temperature

## Abstract

The casting magnesium alloy AZ91D cannot be extruded at room temperature. This paper presents a process for extruding internal threads using AZ91D heated by electromagnetic induction. The feasibility of the process is verified by finite element simulation and experiments. Using DEFORM-3D to simulate the process of extruding a M12 × 1.25 mm threaded hole by electromagnetic induction-assisted heating, the equivalent stress-strain and material flow law in the process of thread deformation was analyzed and verified by experiments. Three parameters—hole diameter, machine speed and heating temperature—were considered to study the influence of different process conditions on the forming torque. The results show that a heating temperature above 523 K can improve the plasticity of AZ91D. The hole diameter has an important influence on the forming torque. The forming process is not suitable for high-speed machining. The surface metal of the thread formed by this process has a strong deformation layer, which can improve the strength and hardness of the thread.

## 1. Introduction

With the development of science and technology in the 21st century, light alloy forming and manufacturing technology has made a great contribution to the development of aerospace, automobile, major equipment, energy, weapons, shipbuilding and information industries [1]. At present, light alloys mainly include aluminum, magnesium, titanium and other high-performance light alloys. Magnesium alloy is the lightest structural metal, with a density two thirds that of aluminum and one quarter that of iron. It has high strength, rigidity, damping, machining and thermal conductivity, and a high absorption of vibration and impact. It is used to improve resource optimization and emission reduction in the field of traffic engineering. Magnesium alloy is lighter, more environmentally friendly and safer. For ensuring automobile safety, the effect of magnesium alloy on reducing automobile mass and energy consumption is particularly significant. Magnesium and its alloy are widely used in automobile manufacturing enterprises. Magnesium alloy is used in the automobile industry instead of steel in engines, saving 0.25l/100 km fuel in carbon dioxide emissions, making the center of gravity of the automobile move backward, and improving the steering performance of the automobile [2].

In the field of machinery, the screw connection is the most common connection mode in the assembly of parts, which is widely used in various industrial fields. In the machining process, 60% of the holes are machined, of which nearly 50% are threaded holes [3]. The threaded connection can be assembled and disassembled quickly and accurately many times, while ensuring that the parts connected with each other will not be damaged [4].

When traditional cutting tap is used to tap magnesium alloy, chips will be produced in the process of machining. It is difficult to clean a chip stuck on the inner wall of the thread, which affects the cleanliness of the product. In the processing and use of threaded holes, the phenomenon of broken teeth and sliding wires is common, which seriously affects the assembly quality [5]. Therefore, in order to ensure that the thread will not be damaged when it is loaded and unloaded many times, the threaded hole to be processed must have high strength and rigidity. In this case, the technology of cold extrusion to process the threaded hole was born. In recent years, cold extrusion threading is becoming more and more important in the manufacturing industry [6]. With the improvement of processing environments and the concept of “green manufacturing”, the application of extrusion threading in industry is increasing. As the tool life is prolonged, the machining accuracy, surface roughness, mechanical strength and reliability of the thread are improved, the process cleanliness is improved, and the production cost is reduced, so from an economic point of view, the extrusion of internal threads is also very important. The biggest advantage of the extrusion of the thread is that the thread is formed by plastic deformation, which not only has good deformation ability but also can maintain high mechanical strength [7,8]. Therefore, compared with the traditional tapping process, extrusion tapping is a good choice.

Most of the research on thread formation focuses on the processing technology. By analyzing the variables in the process, the complete thread profile is obtained. Carvalho et al. [9] used factorial design and variance analysis to optimize the process parameters for AM60 magnesium alloy cold extrusion internal threads, and analyzed the influence of various factors such as initial hole diameter, forming speed and tool type on the thrust, torque, filling rate and hardness in the forming process. The goal was to reduce the bifurcation wave peak of the internal thread and finally obtain a better thread profile quality. Dias [10] proposed a special process for machining magnesium alloy internal threads, analyzed the influence of forming speed and screw feeding rate on the forming process, and found that an increase in the screw feeding rate reduced the torque and thrust during the processing. Sérgio [11] used coated and uncoated tools to extrude the internal thread of 7075 aluminum alloy, analyzed the causes of the formation of the burr at the inlet and outlet of the thread, and then optimized the process parameters in the extrusion process. The results show that a change in the initial diameter only affects the formation of the entrance burr, and the forming speed is the most important factor affecting the formation of the exit burr. Pereira [12] studied the influence of each variable in the two manufacturing processes of cutting threads and extrusion thread formation on the forming process, and determined the rules of the influence of each cutting edge of the tool on the torque, and the compensation effect of a new fixture system on the synchronous error.

There is little research on the three-dimensional finite element analysis of the extrusion thread forming process. Domblesky and Feng [13,14] established two-dimensional and three-dimensional models of screw rolling using “deform 3D”, Warrington et al. [15] established a finite element model under deform three-dimensional software, an equivalent tapping to linear scratch experiment to study the formation of split peaks in the tapping process, and then improved the tapping design and verified their models with experiments. Mathurin et al. [16] used the three-dimensional finite element model of 45 degree sector (discrete rigid screw body) to determine the displacement and thread shape during the forming process. They also conducted parametric studies to determine the most influential process parameters. The results show that the diameter of the guide hole has an important influence on the tightening torque. Dinger [17] used the method of numerical simulation and experiments to analyze the assembly process of screw thread formation. Through parametric study, the influence of the friction conditions and hole diameter on the tightening torque was determined, and it was concluded that the hole diameter has an important influence on tightening torque.

Most of the magnesium alloys have a closely packed hexagonal crystal structure with low symmetry. Their axial ratio is 1.632, which is close to the ideal packed value of 1.633. There are few slip systems at room temperature, and it is difficult to cold work [18]. Among many magnesium alloy materials, cast magnesium alloy AZ91D is one of the most widely used magnesium alloy materials, which is widely used in the transmission systems, engine systems and chassis systems of automobile. In automobile parts, a thread machined by AZ91D material often has a slip thread and insufficient thread strength, while AZ91D is a cast magnesium alloy, which is difficult to be formed at room temperature and also unable to be processed by extrusion. Electromagnetic induction-assisted heating forming technology can realize the on-line real-time heating of components, and solve the technical problem of too-rapid cooling in the hot forming process for light material components [19]. In this paper, a processing technology for the electromagnetic induction-assisted extrusion of AZ91D internal threads is proposed. The finite element model of electromagnetic induction-assisted extrusion is established by using the DEFORM software (Deform 3D V11, SFTC, Berkeley, CA, USA). The law of stress and strain and material flow in the process of processing is analyzed. The influence of process parameters on torque is discussed, so as to obtain high quality threads.

## 2. Materials and Methods

DEFORM-3D is a powerful process simulation and analysis software. It is a finite element system based on a process simulation system. It could be used to analyze the three-dimensional metal flow in various metal forming processes, providing valuable process analysis data and the relevant material and temperature flows in the forming process. The powerful simulation engine of DEFORM-3D can analyze the large deformation and thermal characteristics of many related objects in the metal forming process. The system integrates the functions of the automatic grid re-division generator and local grid subdivision to generate an optimized grid system, reduce the operation scale and improve the calculation efficiency [20]. As the cold extrusion of internal threads belongs to the plastic forming category of large metal deformation, the plastic deformation is far greater than the elastic deformation, so the rigid plastic finite element method was used for analysis.

### 2.1. Rigid-Plastic Finite Element Mathematical Model

Neglecting the volume force and inertia force, the deformation area of material should meet the balance equation:

(1) $$\sigma_{ij,j}=0$$

where $\sigma_{ij,j}$ is the divergence of the stress tensor.

The speed *ν_i_* of the particle in the stress tensor deformation region needs to satisfy the geometric equation:

(2) $${\dot{\varepsilon }}_{ij}=\frac{1}{2}\left(v_{i,j}+v_{j,i}\right)$$

where ${\dot{\varepsilon }}_{ij}$ is strain rate tensor. According to the Mises yield criterion, the constitutive equation of rigid plastic materials is:

(3) $${\dot{\varepsilon }}_{ij}=\frac{3}{2}\frac{\dot{\bar{\varepsilon }}}{\bar{\sigma }}\sigma_{ij}^{\prime }$$

where $\dot{\bar{\varepsilon }}$ is the equivalent strain rate, $\sigma_{ij}^{\prime }$ is the deviatoric Cauchy stress, and $\bar{\sigma }$ is the equivalent stress.

### 2.2. Electromagnetic Induction Heating Simulation

#### 2.2.1. Geometric Model

**Figure 1:**
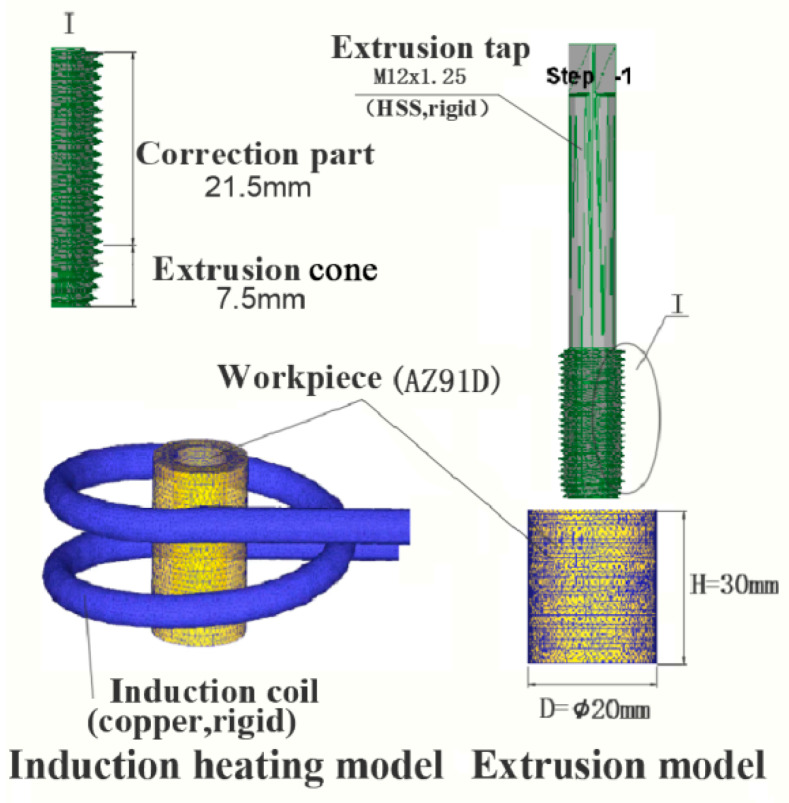
Finite element model.

The research objective of this paper is to study the AZ91D magnesium alloy. The processing of AZ91D magnesium alloy-based extrusion of internal threads is based on high frequency induction heating. Before the processing of the extrusion internal thread, the AZ91D magnesium alloy workpiece is subjected to high frequency induction heating. The finite element model of induction heating is shown in Figure 1.

#### 2.2.2. Workpiece Material Properties Definition

In the simulation of high frequency induction heating, the finite element model is composed of the workpiece and coil. In the process of extruding the workpiece with tap, because of the high strain and strain rate of the workpiece material, the nonlinear characteristics of the material cannot be ignored. The rheological stress constitutive equation applicable to the establishment of the AZ91D magnesium alloy is as shown in Equation (4) [21]:

(4) $$\dot{\varepsilon }=A{\left[\sinh\left(\alpha \sigma \right)\right]}^{n}\times \exp\left(\frac{-Q}{RT}\right)$$

where $\dot{\varepsilon }$ is the strain rate; *A* is the coefficient, *A* = 1.15 × 10^16^ s^−1^; *σ* is the rheological stress; *Q* is the free diffusion activation energy; *α* are the stress level parameters; *n* is the stress exponent; *R* is the gas constant; and *T* is the absolute temperature.

Other parameters of the AZ91D magnesium alloy material are shown in Table 1, and the data generated were added to the deform material library as material properties.

#### 2.2.3. Mesh Generation

The mesh generation of deformable bodies using DEFORM is the basis of numerical simulation analysis. For the subsequent extrusion process for internal threads, which is equivalent to the complex deformation plastic forming numerical simulation problem, it is difficult to simulate the deformation process with only a fixed element mesh. The main reason is that the interference between the extrusion tap boundary and the mesh boundary will seriously distort the finite element mesh, and the distortion will lead to the distortion of the numerical simulation results. Therefore, when the mesh deforms to a certain extent, it is necessary to stop the calculation and adjust and subdivide the mesh. In order to improve the simulation speed and accuracy, mesh subdivision technology was used in the screw forming part. After mesh division, the total number of meshes was 57,951, and the number of cell nodes was 14,703.

#### 2.2.4. Boundary Conditions and Parameters

Since the energized coil provides a magnetic field for the workpiece, the coil was set to Master and the workpiece, to Slave. The current frequency was set to 30,000 Hz, the current density was set to 50 A/mm^2^, and the heating temperature of workpiece was set to 533, 553, 573, 593 and 613 K in the simulation.

### 2.3. Numerical Simulation of Internal Thread Extrusion

#### 2.3.1. Geometric Model and Settings

On the basis of high frequency induction heating, the movement simulation analysis of the internal thread of the workpiece was carried out. The Creao software was used to model the extrusion tap and generate an “STL” file, which was then imported into DEFORM-3D preprocessing for simulation analysis. The finite element model for the extrusion of the internal thread is shown in Figure 1. The structure of the extrusion tap includes a working part and clamping part, and the working part includes an extrusion tap and a correction part. The internal thread extrusion test tap adopts an M12 × 1.25 hexagonal high-speed steel fine thread ordinary fine shank tap, the length of the extrusion tap is 7.5 mm, and the length of the correction part is 21.5 mm.

#### 2.3.2. Establishment of Motion Model

The internal thread extrusion is completed in the machining center. In the actual machining, the main shaft of the machining center drives the tap to rotate at a certain speed, and the internal thread extrusion is completed according to a certain feed rate. In the DEFORM software, the tap was set as a rigid body, and the rotation speed of the machine tool in the actual processing was transformed into the axial speed and rotation angle speed when the tap was extruded. According to the pitch of the formed thread, the relationship between the machine speed and the axial speed and angular speed of the tap in the simulation was as follows:

(5) $$f\;=\;\frac{Pn}{60\;\times \;1000}$$

(6) $$\omega \;=\;\frac{n\pi }{30}$$

where *f* is the axial speed of the tap (m/s), *n* is the machine speed (rpm), *P* is the pitch of the extrusion thread (mm), and *ω* is the Rotation angular velocity of the tap (rad/s).

#### 2.3.3. Setting the Relationship between Objects

Friction is a common and complicated problem in metal forming. The proper treatment of friction boundary conditions and reasonable selection of the friction model directly affect the accuracy of finite element calculation. The inside screw extrusion forming process was modeled by taking into account the high contact stress of the plastic forming process, so the general Coulomb friction model for the extrusion tap edge state of the tooth profile and the extrusion friction between parts is no longer applicable for the extrusion tap part and the plastic deformation of the workpiece contact area for the shear friction model, as shown in Equation (7). According to the characteristics of this process, induction heating-assisted forming and no lubricating fluid are used, and the friction factor was set to 0.7. The heat conduction between the extrusion tap and the magnesium alloy is expressed by the thermal conductivity, which was set to 72 W·M^−1^·K^−1^.

*τ_f_ = mk* (7)

where *τ_f_* is the frictional stress; m is the friction factor, 0 ≤ *m* ≤ 1; and *k* is the shear yield limit, $k=\bar{\sigma }/\sqrt{3}$.

In the process of finite element simulation, only the increase in the slip system for the magnesium alloy at 498 K was considered. The temperature dependence of the mechanical properties was not considered.

#### 2.3.4. Setting of Boundary Conditions

According to the actual processing conditions, the workpiece was fixed on the worktable of the machining center, so all six degrees of freedom on the outer surface of the workpiece were fixed, and the temperature field was added to the workpiece. In the process of simulation, volume compensation was needed to prevent large deviations between the mesh volume and the workpiece volume after mesh division, which would result in large errors in simulation and inaccurate experimental data.

#### 2.3.5. Setting of Simulation Control Parameters

Simulation control parameter setting includes setting the simulation type, step control, stop control, etc. In this work, the Lagrangian incremental method was selected for iterative control and calculation. The simulation type was set to forming mode, which means that the whole machining process only took plastic forming instead of cutting forming; the total number of simulation steps was set to 380 steps; and the data were stored once per step, which was set to 0.04 mm in the solution step definition, that is, one third of the minimum mesh size, and the others were selected as default values.

### 2.4. Experimental Method

**Figure 2:**
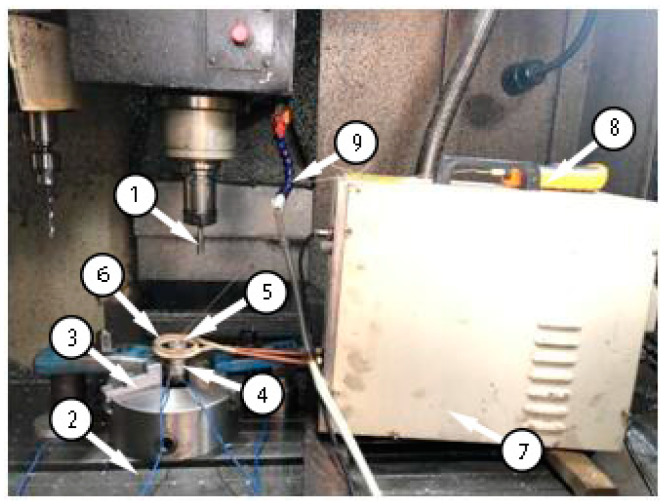
Experimental apparatus for the forming process. 1—Extrusion tap; 2—Worktable; 3—Special fixture; 4—Torque sensor; 5—Temperature sensor; 6—Coil; 7—Induction heater; 8—Thermometer; 9—Cooling water pipe.

In the experiment, the DHK32 machining center was used to carry out the auxiliary heating extrusion processing of the M12 × 1.25 mm internal thread. The experimental device used to study the forming process is shown in Figure 2. The extrusion tap, 1, was installed in the spindle box of the machining center, and the magnesium alloy workpiece was installed in the special clamp, 3, and fixed on the workbench, 2, of the machining center. The induction coil, 6, was placed on the outside of the workpiece, and a 2 cm gap was maintained with the outer circle surface of the workpiece. The water pipe, 9, was cooled with cold water, and the relevant parameters for induction heating with the induction heater, 7, were adjusted. The workpiece temperature was measured with the temperature sensor, 5, and read on the thermometer, 8. The experimental parameters were the same as those of the finite element simulation.

The torque sensor was used to measure the torque produced in the process of internal thread extrusion. The torque sensor consisted of a measuring bridge, an amplifier and an interface circuit composed of a resistance strain gauge (BF-350HA).

After cutting the extruded magnesium alloy internal thread sample, the longitudinal section of the thread tooth was taken for grinding and polishing. The shape of thread was observed using an OLYMPUS-GX71-6230A metallographic microscope (OLYMPUS, Tokyo, Japan).

An HXD-1000 microhardness tester (BAHENS, Shanghai, China) was used to test the microhardness of the samples. Before the test, the sample should be polished in accordance with the requirements of metallographic observation. The test parameters were: load, 50 g; pressure holding time, 20 s.

**Figure 3:**
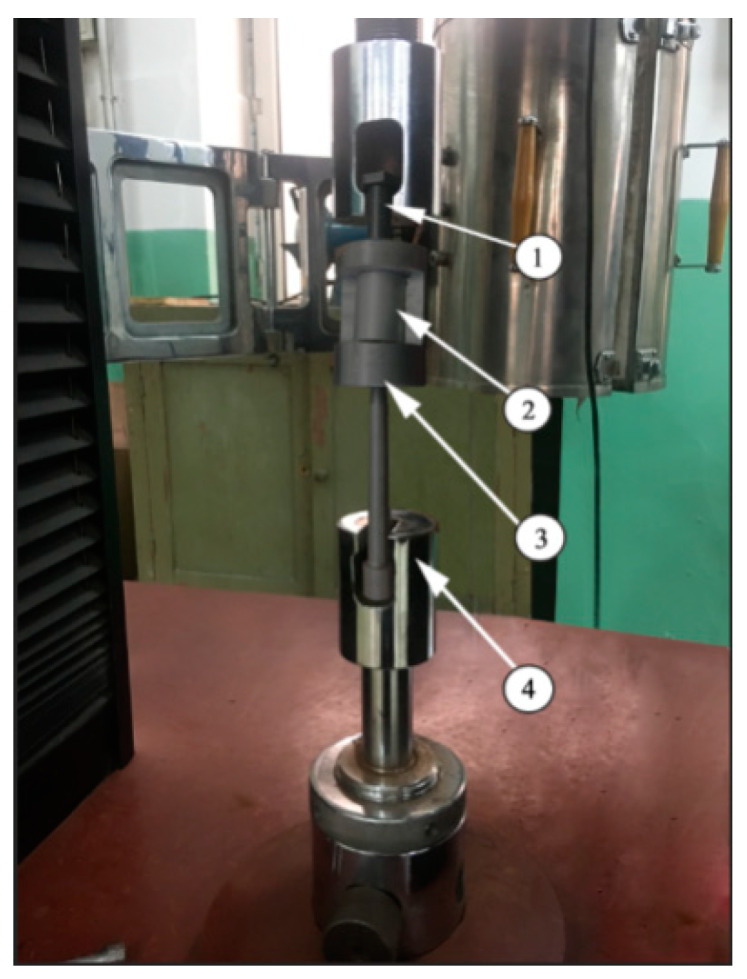
Tensile test device. 1—M12 × 1.25 screw; 2—Magnesium alloy; 3—Special tooling; 4—Cupping machine.

Through a quasi-static tensile test, the tensile strength tests for the cutting thread and cold extrusion thread formation were carried out respectively. As shown in Figure 3, the magnesium alloy, 2, was put into the special tooling, 3, and the magnesium alloy from the top of the special tooling was screwed in with an M12 × 1.25 standard screw. They were put together on the cupping machine, 4, for the destructive test. The specifications of each thread are M12 × 1.25 mm, the screw length is 18 mm, and the tensile force values are the average values of six threads. The experimental results are all thread pull off values, and the maximum tensile force value is obtained.

## 3. Results and Discussion

### 3.1. Results and Discussion of Finite Element Simulation

#### 3.1.1. Equivalent Stress and Strain in the Forming Process

**Figure 4:**
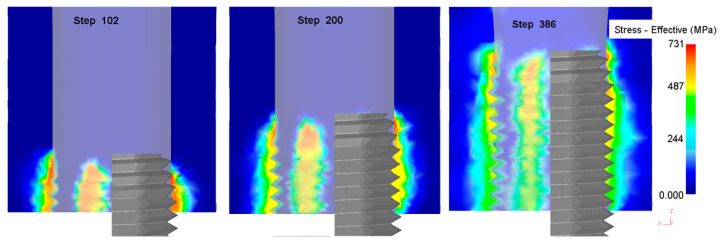
Equivalent stress nephogram of different steps.

**Figure 5:**
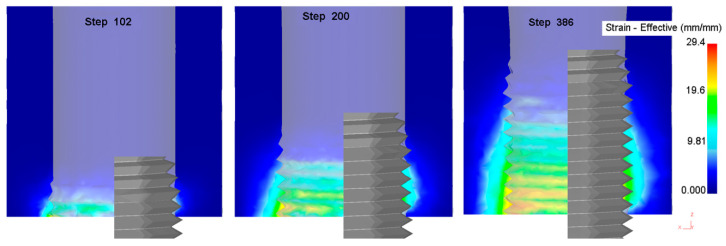
Equivalent strain nephogram of different steps.

The equivalent stress and strain in the metal forming process is the physical quantity by which to judge the degree of metal deformation. Figure 4 shows the change in the equivalent stress in the process of thread formation. With an increase in the processing depth of the extrusion tap, the equivalent stress decreases gradually, and the maximum stress occurs in the area where the extrusion tap part of the extrusion tap interacts with the workpiece. Figure 5 shows the change in the equivalent strain during the process of thread formation. With an increase in the processing depth of the extrusion tap, the equivalent strain increases gradually, and the maximum strain occurs in the area where the correction part of the extrusion tap interacts with the workpiece.

The results for equivalent stress and strain show that the forming process is closely related to the structure of the extrusion tap. A structure with a taper angle is made at the tap part of the extrusion tap, such that the inner hole gradually forms a tooth shape, resulting in a large interaction force between the tap and the workpiece; the correction part is to guide and correct the formed thread shape so that the material is filled with the edge teeth of the extrusion tap, so the interaction force between the tap and the workpiece is small but the metal material displacement increases.

#### 3.1.2. Law of Metal Flow

**Figure 6:**
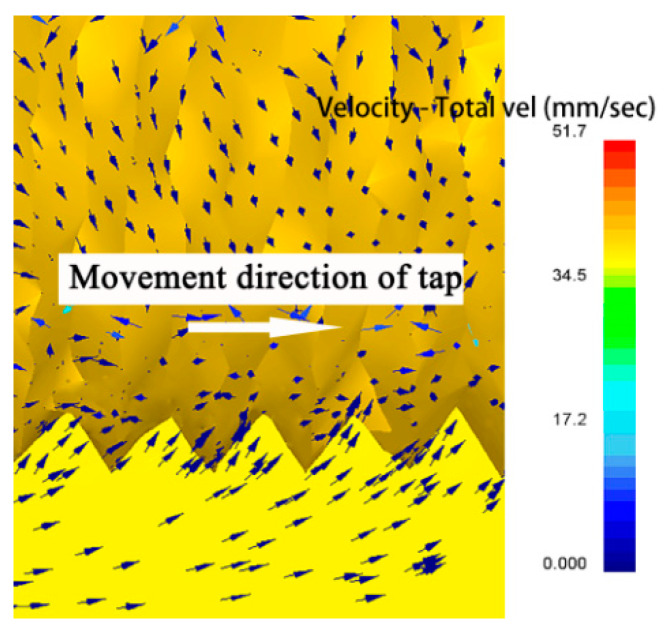
Velocity field distribution in the forming process.

When the extrusion tap was screwed into the diameter of the hole of the workpiece, the edge teeth of the extrusion tap gradually extruded the metal materials around the hole and caused plastic deformation in the limited space. Figure 6 shows the velocity field when the correction part of the extrusion tap acted on the magnesium alloy. It can be seen that the direction of metal flow was basically upstream along the tooth, from the bottom to the top of the tooth.

**Figure 7:**
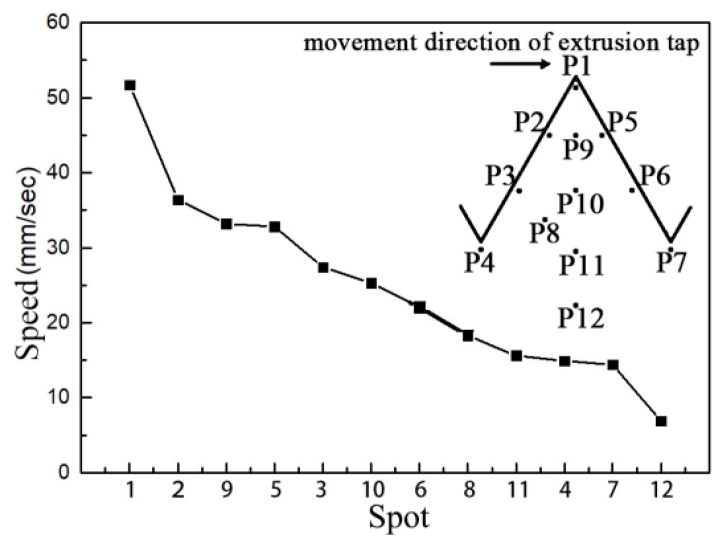
Velocity profile of each point on the thread surface.

In order to quantitatively study the change of speed in the thread profile, 12 points on the profile were taken as shown in Figure 7, and the corresponding curve was drawn by using the point tracking function in the post-processing of deform. It can be seen from the figure that the speed of the P1 point at the crown is the largest. On the whole, the speed of a point closer to the crown was greater than that of a point closer to the bottom; P2, P5 and P9 were greater than P4, P7 and P11; and the speed of P12 at the bottom was the smallest. At the same time, it was found that the velocities of P2, P3 and P4 upstream at the same height were higher than those of P5, P6 and P7 on the right.

It can be seen from the results in Figure 6 and Figure 7 that when the extrusion tap interacted with the workpiece, the metal flow followed the law of minimum resistance, and the groove of the edge teeth of the extrusion tap with the minimum flow resistance would be extruded. The movement direction of the extrusion tap would make the flow resistance of metal particles on both sides of the thread profile different; the movement speed of metal particles upstream in the thread profile is higher than of those downstream.

#### 3.1.3. Influence of the Hole Diameter

**Figure 8:**
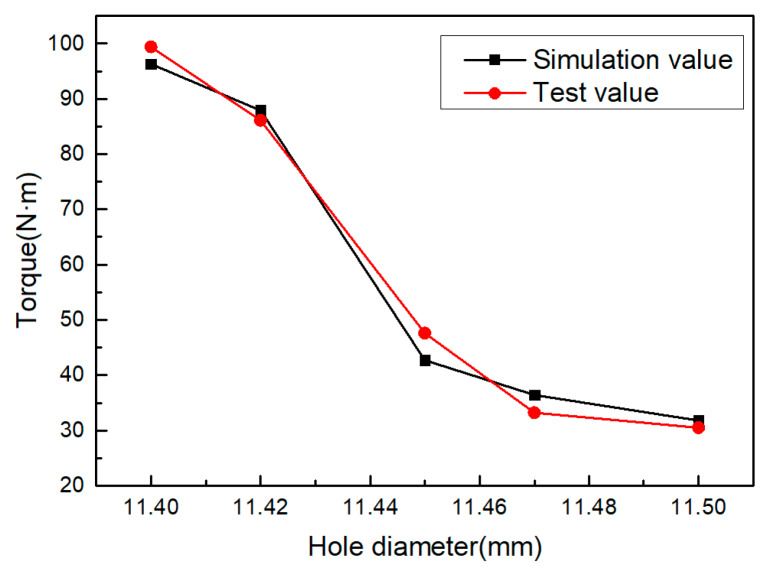
Effect of hole diameter on torque.

When extruding the internal thread with the extrusion tap, the area where the diameter of magnesium alloy workpiece material is larger than the diameter of the hole flows to the area with the smaller diameter, and the diameter of the guide hole significantly affects the shape of thread, thus affecting the torque in the process of thread formation [16]. During the simulation and experiment, the rotating speed of the machine tool was 100 rpm, the auxiliary heating temperature was 573 K, and the diameter of the hole was set to 11.40, 11.42, 11.45, 11.47 and 11.50 mm, respectively. The torque simulation value and torque test value were compared. Figure 8 shows the change of torque under different hole diameters. With an increase in hole diameter, the torque gradually decreased. When the hole diameter changed from Φ11.40 to Φ11.45 mm, the effect of this reduction was more obvious.

The influence of hole diameter on the forming torque shows that the increase in torque is due to the decrease in the strain space of the metal material in the process of extrusion. This is because the extruded workpiece material is constantly filled with the range of the edge teeth of the extrusion tap. The diameter of the hole is large, there are few magnesium alloy materials that can be accommodated in the edge teeth of the extrusion tap, the strain space of the metal material increases, the material deformation is small, and the torque decreases.

#### 3.1.4. Influence of the Hole Diameter

**Figure 9:**
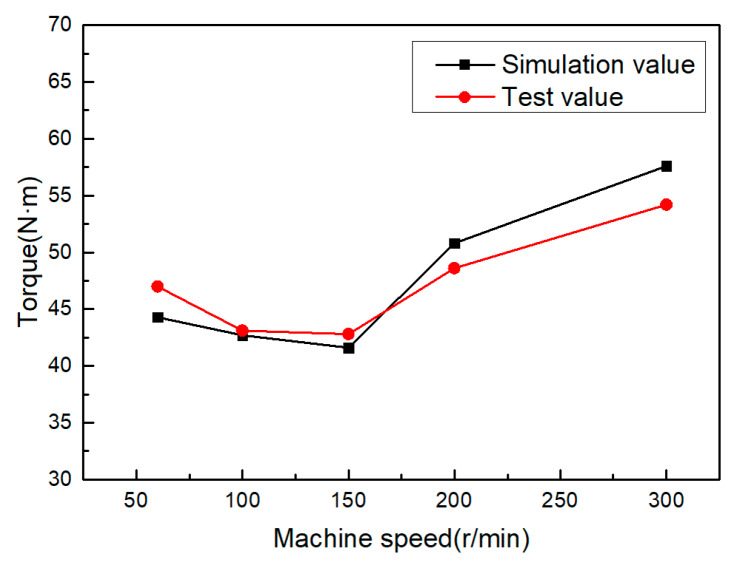
The effect of machine speed on torque.

One can compare the torque simulation value with the torque test value under the conditions of a bottom hole diameter of 11.45 mm, an auxiliary heating temperature of 573 K, and a machine speed of 50, 100, 150, 200 and 300 rpm, respectively. Figure 9 shows the change curve for the maximum torque random machine speed. It can be seen from the figure that the maximum torque in the internal thread forming process first decreases with the increase in the random machine speed, but the decrease trend is very small, and then increases with the increase in the machine speed. When the machine speed is 150 rpm, the extrusion torque is at its minimum.

From the influence of machine speed on forming torque, it can be seen that when the machine speed is very low—given the fact that the contact time between the workpiece and the extrusion tap is longer and that the temperature of the extrusion deformation area is decreased—the metal yield strength is increased and the extrusion pressure is increased. Because the auxiliary heating forming is adopted in this process, the influence of temperature on the deformation zone is not obvious at this point, and the trend of reduction is not obvious. When the machine speed of machine tool reaches 300 rpm, the machine speed is too fast, the material of workpiece has not been fully plastically deformed before the contact time period, and then the extrusion process of the next tap edge is completed. Therefore, at this point, the deformation of the material is not sufficient so the torque in the forming process will be increased.

#### 3.1.5. Auxiliary Heating Temperature

**Figure 10:**
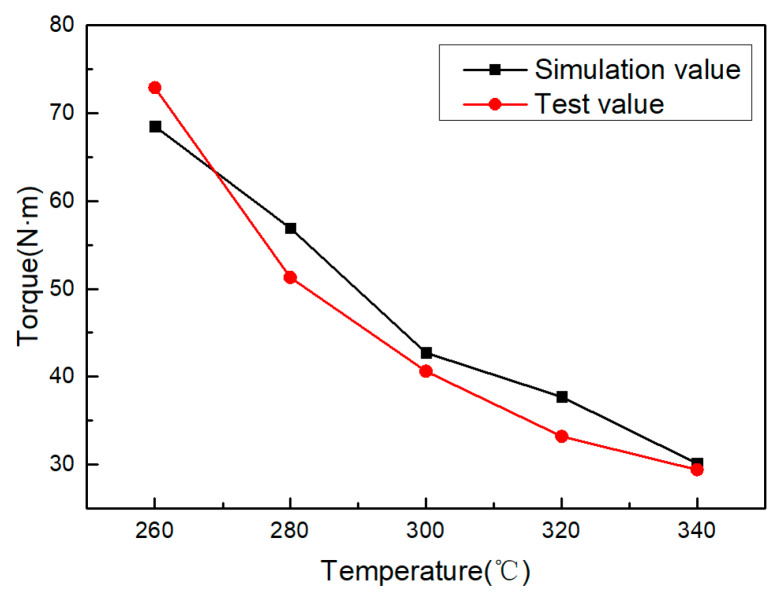
Effect of heating temperature on torque.

Temperature is the key factor affecting the plastic deformation ability of the magnesium alloy, and it is also an important parameter of this process. The deformation behavior of the magnesium alloy at 523–773 K and a 10^−4^–10^−2^ s^−1^ strain rate can be divided into three temperature zones of lower than 523, 523–623 and 623–773 K according to the deformation characteristics [22]. The heating temperature of the magnesium alloy in this process was set between 523–623 K, at 533, 553, 573, 593 and 613 K, respectively. The diameter of hole for other parameters was 11.45 mm, and the rotating speed of the machine tool was 100 rpm. Figure 10 shows the effect of heating temperature on the torque. As the temperature increases, the workpiece material softens due to recovery, reducing the torque in the forming process.

When the temperature of the magnesium alloy is lower than 498 K, there are only three geometrical slip systems and two independent slip systems [22]. When the temperature is higher than 498 K, the increase in temperature will increase the amplitude of the atomic vibration, activate the potential slip surface and slip direction, and start the additional angle cone slip surface; at the same time, the softening caused by recovery will also make the magnesium alloy have a higher plasticity. When the plasticity of the magnesium alloy material is improved, the stress produced in the metal during the processing will be smaller and the deformation resistance will be reduced, so the torque will be reduced.

### 3.2. Experimental Results and Discussion

#### 3.2.1. Microstructure

**Figure 11:**
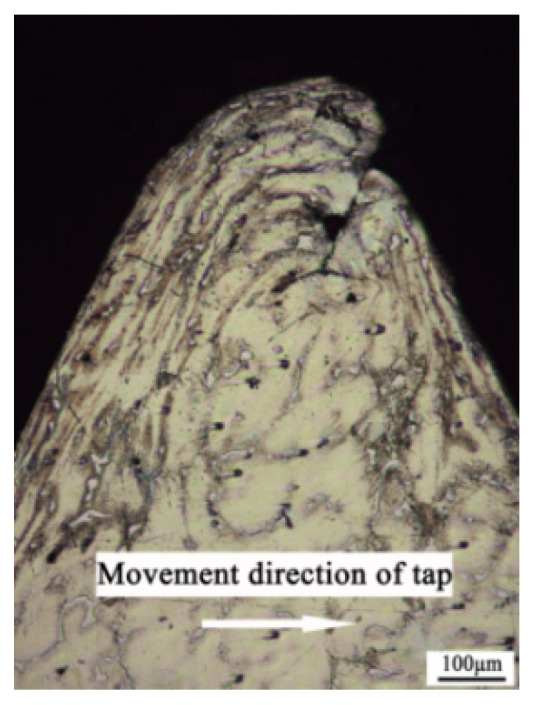
Optical image of the thread profile.

Figure 11 shows the optical microscopic image of the cross section of the thread profile manufactured with the hole diameter being 11.45 mm, the machine speed being 100 rpm and the heating temperature being 573 K. It can be seen from the figure that during the forming process, plastic flow occurs in the material, a streamline is formed in the metal fiber structure of the thread surface layer, and the flow trend is basically consistent with that shown in Figure 6.

From the optical image of the thread profile, it can be seen that the surface metal flows continuously along the deformation direction to the extrusion tap groove with little resistance, and the extrusion tap groove is filled with metal continuously, thus forming the entire thread profile with the continuous extrusion. The plastic deformation of the metal is the most severe in the surface layer; the deeper the distance from the surface layer, the smaller the deformation. After the plastic deformation of the surface metal, the shape of the grain is obviously flattened and drawn into a thin strip, forming a fiber structure. Due to the formation of these fibers, the strength and hardness of the thread can be improved.

#### 3.2.2. Microhardness

**Figure 12:**
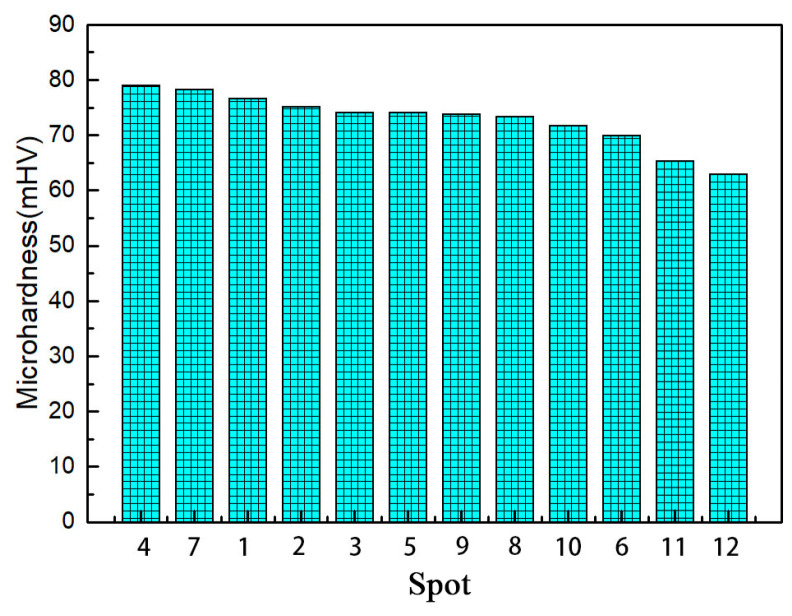
Microhardness of each point on the thread profile.

In the process of thread processing, the use of an extrusion tap led to a plastic flow of the workpiece material, and the auxiliary heating temperature did not exceed the recrystallization temperature, which inevitably induced the strain hardening of the material. This strain hardening can be measured by microhardness. The test points of each sample are shown in Figure 7. Then, a minimum value and a maximum value were removed for each sample, and the average hardness values of the remaining three different parts were taken for recording. Figure 12 shows the hardness value with a hole diameter of 11.42 mm, machine speed of 100 rpm and heating temperature of 533 K. The initial hardness of the workpiece material is 62.9 mHV. It can be seen from Figure 12 that the hardness values of points P1–P11 are higher than those of point P12, which indicates that the hardening of materials occurs on all surfaces of the formed thread surface. The hardness values of point P1 at the bottom of the thread are the largest, and the hardness values of point P1 at the top of the thread are higher than those of the points on the two sides. It is also found that the hardness values of points P2 and P3 upstream are higher than those of points P5 and P6.

The microhardness measurement results show that the thread obtained by this process has undergone strain hardening and that there is a strong deformation layer on the thread surface. The hardness value at the root of the thread is the largest, which is the area with the most serious deformation of the thread surface. The main reason is that the edge teeth of the extrusion tap directly act on the bottom of the thread, making the bottom metal first meet the yield criterion and enter the plastic state. The hardness value on the upstream of the thread profile is greater than that on the downstream, because of the contact between the upstream part of the thread and the surface of the tap. The stress is greater than in the profile on the downstream part, and the movement direction of the tap will drive the material to move to the top of the thread, resulting in material accumulation. From the analysis of the hardening of a thread surface, it is found that the speed change of 12 points is basically the same as that in the previous simulation.

#### 3.2.3. Tensile Strength

**Figure 13:**
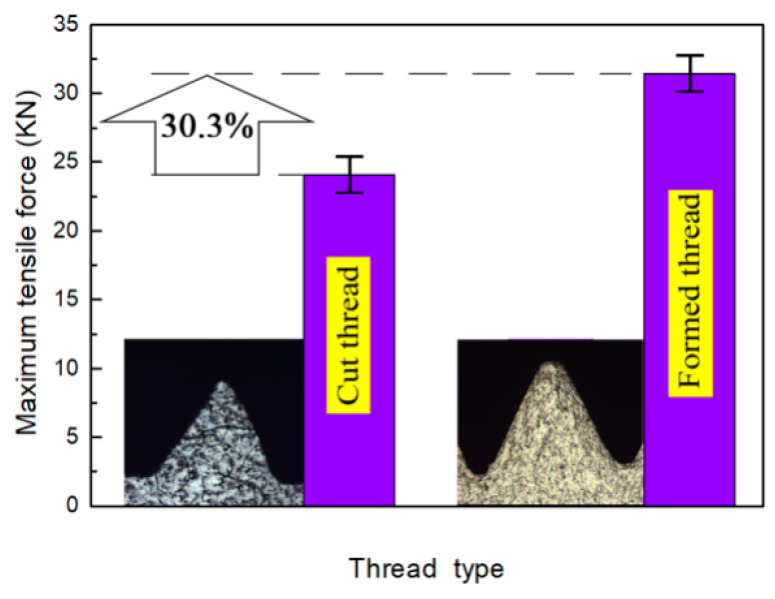
Comparison of the maximum tensile force of the thread with different processing methods.

The basic characteristic of a threaded hole is strength. Through tensile tests, the thread characteristics obtained by cutting and auxiliary heating extrusion were quantitatively compared. Figure 13 shows the maximum tensile forces of cut thread and formed thread with the same hole diameter of 11.45 mm. The figure shows that the maximum tensile force of cut thread is 24.1 kN, and the maximum tensile force of formed thread is 31.4 kN, which is 30.3% higher than that of cut thread.

Because of the plastic flow in the forming process for extrusion thread, the structure of the thread profile is continuous, and there is a deformation layer in the thread. It is this strain hardening that causes the strength of the extrusion thread to increase.

## 4. Conclusions

In this paper, an extrusion-based process for forming AZ91D magnesium alloy internal threads is proposed. The key point of the process is to use an electromagnetic induction coil to assist in heating the magnesium alloy, so that the plasticity of the cast magnesium alloy can be improved compared with that made at room temperature.

Through the simulation and analysis of the equivalent stress-strain and the law of metal flow in the forming process, it was found that the structure of the extrusion tap is also a relevant factor affecting the internal thread formation. The moving direction of the extrusion tap would cause different degrees of material accumulation on both sides of the thread profile.

The heating temperature is a key parameter in this process. The temperature range makes the deformation resistance of metal lower than that with cold working, the energy consumption less than that with cold working, and metal plasticity larger than that with cold working. With an increase in temperature, the yield strength of the metal decreases and the torque of the forming process decreases.

The hole diameter is an important factor in the extrusion internal thread formation process. With a decrease in the hole diameter, the torque increased, especially when the diameter of the hole changed from 11.45 to 11.40 mm.

This process is not suitable for high-speed machining. When the machine speed exceeds 200 rpm, the deformation of the workpiece material is not sufficient, resulting in an increase in the torque.

Strain hardening is the root cause of internal threads in extrusion, which is caused by the plastic flow of metal under the interaction of the extrusion tap and workpiece. The hardened layer formed on the thread surface will improve the mechanical properties of the thread. The maximum tensile force of the thread formed by this process is 30.3% higher than that formed by thread cutting.

The process in this paper can be applied to the extrusion processing of other alloy materials with poor plasticity. Future research should take into account the optimization of process parameters and more complex friction and contact models.

## Figures and Tables

**Table 1:** Basic parameters of the AZ91D magnesium alloy at room temperature.

Young’s Modulus (GPa)	Poisson Ratio	Density (g∙cm^−3^)	Tensile Strength (MPa)	Yield Strength (MPa)	Coefficient of Thermal Expansion (K^−1^)	Thermal Conductivity (W∙M^−1^∙K^−1^)	Heat Capacity (J∙g^−1^∙K^−1^)	Radiation Coefficient
45	0.35	1.82	250	160	26	72	1.02	0.7
